# Local confinement of disease-related microbiome facilitates recovery of gorgonian sea fans from necrotic-patch disease

**DOI:** 10.1038/s41598-018-33007-8

**Published:** 2018-10-02

**Authors:** Elena Quintanilla, Catalina Ramírez-Portilla, Boahemaa Adu-Oppong, Gretchen Walljasper, Stefanie P. Glaeser, Thomas Wilke, Alejandro Reyes Muñoz, Juan A. Sánchez

**Affiliations:** 1 Department of Animal Ecology and Systematics, Justus Liebig University Giessen, Giessen, Germany; 2 Department of Biological Sciences, Laboratory of Marine Molecular Biology (BIOMMAR), Universidad de los Andes, Bogotá, Colombia; 3 Department of Biological Sciences, Research Group on Computational Biology and Microbial Ecology (BCEM), Universidad de los Andes, Bogotá, Colombia; 4 Center for Genome Sciences and Systems Biology, Washington University School of Medicine, St Louis, Missouri USA; 5 Institute of Applied Microbiology, Justus Liebig University Giessen, Giessen, Germany; 6 Max Planck Tandem Group in Computational Biology, Universidad de los Andes, Bogotá, Colombia

## Abstract

Microbiome disruptions triggering disease outbreaks are increasingly threatening corals worldwide. In the Tropical Eastern Pacific, a necrotic-patch disease affecting gorgonian corals (sea fans, *Pacifigorgia* spp.) has been observed in recent years. However, the composition of the microbiome and its disease-related disruptions remain unknown in these gorgonian corals. Therefore, we analysed 16S rRNA gene amplicons from tissues of healthy colonies (n = 19) and from symptomatic-asymptomatic tissues of diseased colonies (n = 19) of *Pacifigorgia cairnsi* (Gorgoniidae: Octocorallia) in order to test for disease-related changes in the bacterial microbiome. We found that potential endosymbionts (mostly *Endozoicomonas* spp.) dominate the core microbiome in healthy colonies. Moreover, healthy tissues differed in community composition and functional profile from those of the symptomatic tissues but did not show differences to asymptomatic tissues of the diseased colonies. A more diverse set of bacteria was observed in symptomatic tissues, together with the decline in abundance of the potential endosymbionts from the healthy core microbiome. Furthermore, according to a comparative taxonomy-based functional profiling, these symptomatic tissues were characterized by the increase in heterotrophic, ammonia oxidizer and dehalogenating bacteria and by the depletion of nitrite and sulphate reducers. Overall, our results suggest that the bacterial microbiome associated with the disease behaves opportunistically and is likely in a state of microbial dysbiosis. We also conclude that the confinement of the disease-related consortium to symptomatic tissues may facilitate colony recovery.

## Introduction

Corals worldwide are severely threatened by the increased incidence of diseases over recent decades^1,2^. As an important modifying factor of reef systems, diseases may reduce coral cover, decrease diversity and affect coral life-history traits^3^. As a consequence, changes in coral communities and massive coral die-offs have occurred globally, challenging the resilience of coral ecosystems^4–6^.

Corals are meta-organisms comprising the coral animal itself and a microbial community (the ‘microbiome’) such as protists, bacteria, archaea, viruses and fungi, which collectively constitute the coral holobiont^7,8^. The microbiome confers benefits to the holobiont including nutrient acquisition and disease resistance through the production of antibiotic compounds^9,10^. Indeed, metabolic complementation (i.e. production of metabolites by each partner that are crucial for the survival of other’s holobiont partners) highlights reciprocal relationships existing between microbiome and coral host^11^. Identifying the stable and consistent components occurring within the coral microbiome, the ‘core microbiome’, provides insights into the functions that these assemblages offer to the holobiont and enables the understanding of critical microbiome changes associated with disturbances^12,13^. Such changes in the composition and function of the microbiome affect holobiont fitness and disease susceptibility, potentially leading to disease outbreaks^1,14^.

Understanding coral disease causation remains challenging as complex interactions exist between causative agents, environment and host^1,15^. External factors such as infectious or opportunistic pathogens and environmental stressors may affect a compromised coral holobiont, potentially triggering diseases^15^. In particular, complex interactions have been identified within coral microbial communities driving disease processes^16^. The latter may explain why the etiology of most coral diseases cannot be attributed to single causative agents and hence, terms such as ‘opportunistic pathogens’, ‘polymicrobial diseases’, ‘pathobiome’ and ‘dysbiosis’ (i.e. microbial imbalance) have been associated with coral diseases^16–18^.

Healthy and disease-related coral microbiomes are poorly understood at an intra-colony level^19^. Different bacterial assemblages may occur within diseased colonies, revealing intermediate health states^20^. Therefore, studies of tissues differentially affected within diseased colonies may promote a better understanding of the spatial effects of the disease-associated microbiome and disease progression, which in turn may allow for the identification of different coral health states.

Die-offs related to disease outbreaks have been observed in the gorgonian sea fan *Pacifigorgia cairnsi* (Gorgoniidae: Octocorallia)^21,22^. This species is native to the TEP and dominates the infralitoral seascape at Malpelo Island (Colombian TEP), forming dense aggregations on rocky outcrops and walls that occur up to 30 m water depth^21,23^. In this study we tested for disease-related changes in the bacterial microbiome of *P*. *cairnsi* sea fans by generating 16S rRNA gene amplicons from healthy (n = 19) and diseased (n = 19) colonies. In order to achieve this goal, we identified the bacterial community composition of the core microbiome associated with the healthy state as a baseline for our subsequent analyses. We then compared tissues from healthy colonies and tissues affected by the disease to assess disease-related shifts in bacterial community compositions and functional profiles. Finally, we tested for disease-related shifts occurring within diseased colonies (i.e. between symptomatic and asymptomatic tissues) in order to identify the relationships between the bacterial microbiome and gorgonian health states at an intra-colony level.

## Results

**Figure 1:**
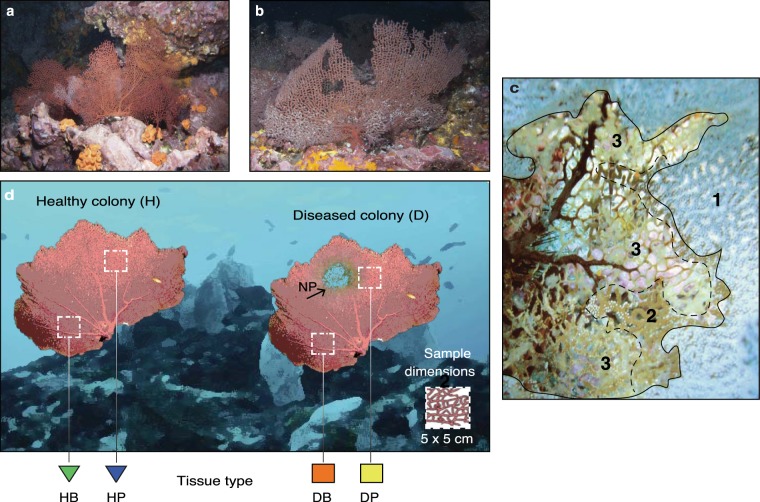
Sampling design of healthy and diseased colonies of *Pacifigorgia cairnsi*. (**a**) Healthy colony and (**b**) diseased colony affected by NPD at Malpelo Island. (**c**) Detail of *P*. *cairnsi* lesion showing tissue with extended polyps (1), tissue with retracted polyps (2) and necrotic areas lacking polyps and coenenchyme (3). (**d**) Diagram showing sampling design. Samples (5 × 5 cm) were taken from peripheral tissue of healthy colonies (HP), basal tissue of healthy colonies (HB), peripheral (symptomatic) tissue of diseased colonies (DP) and basal (but asymptomatic) tissue of diseased colonies (DB). Necrotic patches (NP) were not included in DP samples.

The total count of quality-filtered reads obtained was 3,726,194, with a minimum of 13,117 and a maximum of 103,590 of counts per sample. We decided to normalize at 29,000 reads per sample in order to remove a minimum number of samples from the study and considering the fact that rarefaction curves reached near saturation well before 29,000 reads for the three alpha diversity metrics evaluated (Supplementary Fig. S1). This trend was observed in 73 of the total 80 samples. The alpha diversity metrics of samples from each tissue type at a subsampling of 29,000 reads per sample are summarized in Supplementary Table S1. See Fig. 1 for sampling design.

### Core bacterial microbiome composition of healthy colonies

**Figure 2:**
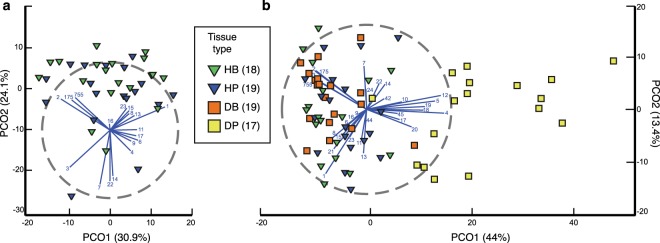
PCoA plots based on a Bray-Curtis dissimilarity matrix of bacterial community compositions in healthy and diseased *P*. *cairnsi* samples. (**a**) Community compositions from samples of healthy colonies (HB and HP) and (**b**) from samples of healthy and diseased colonies (HB, HP, DB and DP) were compared using Bray-Curtis dissimilarity metric on the square-root transformed relative abundances. Note that disease basal (DB) samples showed no symptoms of disease. Principal Coordinate Analysis was used for visualization purposes, and the first two components (explaining over 50% of the variation) are displayed. The number of samples from each type of tissue is indicated within parenthesis. Vectors’ numbers correspond to taxa assigned at OTUs level: (1) genus *Mycoplasma*, (2) genus *Endozoicomonas*, (3) genus *Endozoicomonas*, (4) order Bacteroidales, (5) genus *Aquimarina*, (6) family Oceanospirillaceae, (7) order Alteromonadales, (8) domain Bacteria, (9) order Kiloniellales, (10) class Alphaproteobacteria, (11) class Alphaproteobacteria, (12) genus *Loktanella*, (13) domain Bacteria, (14) family Pirellulaceae, (15) class Alphaproteobacteria, (16) order 34P16, (17) order Kiloniellales, (18) order Oceanospirillales, (19) family Rhodobacteraceae, (20) genus *Polaribacter*, (21) domain Bacteria, (22) genus *Synechococcus*, (23) class Spirochaetes, (24) order CAB-I, (42) genus *Nitrosopumilus*, (44) genus *Vibrio*, (45) species *Polymorphum gilvum*, (175) genus *Endozoicomonas*, (755) genus *Endozoicomonas*.

Bacterial community compositions associated with tissue samples from healthy colonies (HB and HP) did not differ. Non-significant differences were observed in species richness Chao 1, in number of OTUs (operational taxonomic units) and in Shannon diversity between HB and HP samples (n = 37) (Supplementary Fig. S1). Beta diversity analysis, visualized using the first two coordinates of the PCoA plot did not show clusters of samples corresponding to types of tissues (Fig. 2a). Moreover, non-significant differences in community compositions and in the homogeneity of multivariate dispersions were inferred between these two types of tissues (PERMANOVA, *P* > 0.05, Permutational Analyses of Multivariate Dispersions, PERMDISP, *P* > 0.05 Table 1a). Accordingly, the Similarity Percentage (SIMPER) analysis revealed that the same set of OTUs contributed to the homogeneity within each type of tissue (Supplementary Table S2).

**Table 1:** PERMANOVA and PERMDISP analyses.

	Source	df	PERMANOVA				PERMANOVA Pair-wise tests			PERMDISP		
			SS	MS	Pseudo-F	P (perm)	Groups	t	P (perm)	Groups	t	P (perm)
a	Tissue types	1	220	220.260	0.869	0.519				HB, HP	0.932	0.523
	Res	35	8872	253.470			—	—	—			
	Total	36	9092									
b	Tissue types	3	11499	3833	12.368	0.0001*	DP, DB	4.969	0.0001*	DP,DB	2.628	0.024
	Res	69	21384	309.910			DP, HB	4.659	0.0001*	DP,HB	1.754	0.126
	Total	72	32883				DP, HP	4.317	0.0001*	DP,HP	1.229	0.295
							DB, HB	0.982	0.462	DB,HB	0.763	0.486
							DB, HP	1.455	0.026	DB,HP	1.638	0.159
							HB, HP	0.933	0.539	HB,HP	0.709	0.526

(a) Samples from healthy colonies (HB and HP) and (b) samples from healthy and diseased colonies (HB, HP, DB and DP) *P < 0.0083, Bonferroni corrected.

**Figure 3:**
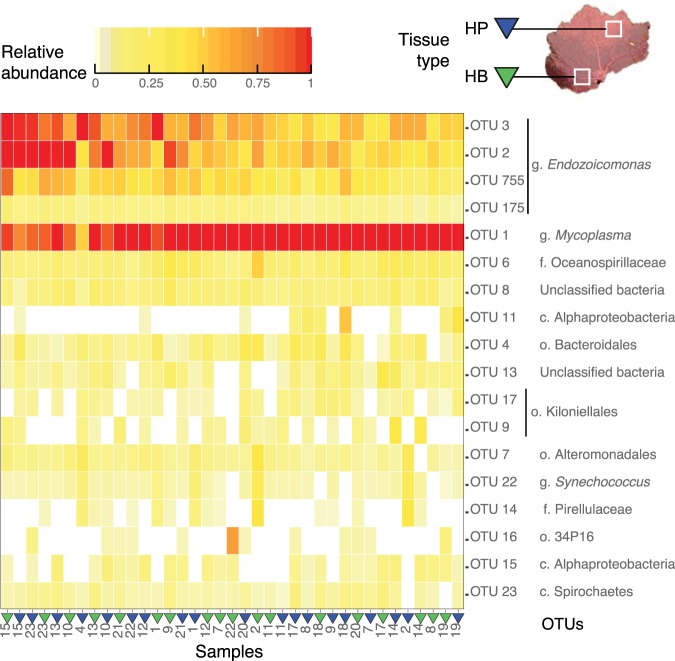
Heatmap of relative abundances of OTUs in tissue samples from healthy colonies. Eighteen OTUs that accounted for 95% of the cumulative abundance in healthy colonies are displayed, with the top seven OTUs corresponding to the core microbiome (g: genus, f: family, c: class, o: order). Samples (columns) were ordered according to their spatial position displayed along the first PCoA component (explaining over 30% of the variation, Fig. 2a).

Given the similarity of the alpha diversity metrics and community compositions of healthy colonies, the core bacterial microbiome of *P*. *cairnsi* was defined as all OTUs present in all healthy samples. Eighteen OTUs accounted for the cumulative 95% of total abundance. From those, seven OTUs comprised the core microbiome, with *Mycoplasma* and *Endozoicomonas* being the prevalent components (Fig. 3). *Mycoplasma* (OTU1) was the most abundant core member (44.87 ± 13.43 average relative abundance given in %), while *Endozoicomonas* was the most diverse bacterial group among the core microbiome with four OTUs: OTU3 (19.69 ± 10.10), OTU2 (17.94 ± 9.72), OTU755 (8 ± 3.80) and OTU175 (0.44 ± 0.20). OTUs assigned to the family Oceanospirillaceae (OTU6, 2.12 ± 1.75) and unclassified bacteria (OTU8, 1.08 ± 0.71) also comprised the core bacterial microbiome. Additionally, some OTUs were present in the majority of the samples (>86%), displaying low relative abundances: Bacteroidales (OTU4, 1.22 ± 1.64), Alteromonadales (OTU7, 0.67 ± 1.30), *Synechococcus* (OTU22, 0.34 ± 0.95), Spirochaetes (OTU23, 0.18 ± 0.26) and unclassified bacteria (OTU13, 0.68 ± 1.27) (Fig. 3).

### Comparison of bacterial community compositions and functional profiles between tissue samples from healthy and diseased colonies

Symptomatic samples (DP) harboured bacterial assemblages different from those present in the asymptomatic samples (HB, HP and DB). The number of observed OTUs, bacterial richness and Shannon diversity within samples were significantly higher in DP compared to HB, HP and DB samples (Supplementary Fig. S1). In the ordination analysis, DP samples clustered apart from HB, HP and DB samples, suggesting different bacterial community composition (Fig. 2b). Moreover, PERMANOVA analyses revealed significant differences in community compositions in pair-wise comparisons between DP samples and samples from each type of the asymptomatic tissues (*P* < 0.0083, Table 1b). The homogeneity observed in multivariate dispersions among the four sampling groups (PERMDISP, *P* > 0.0083, Table 1b) confirmed that the significant differences obtained with PERMANOVA were due to differences in bacterial community compositions^24^.

**Figure 4:**
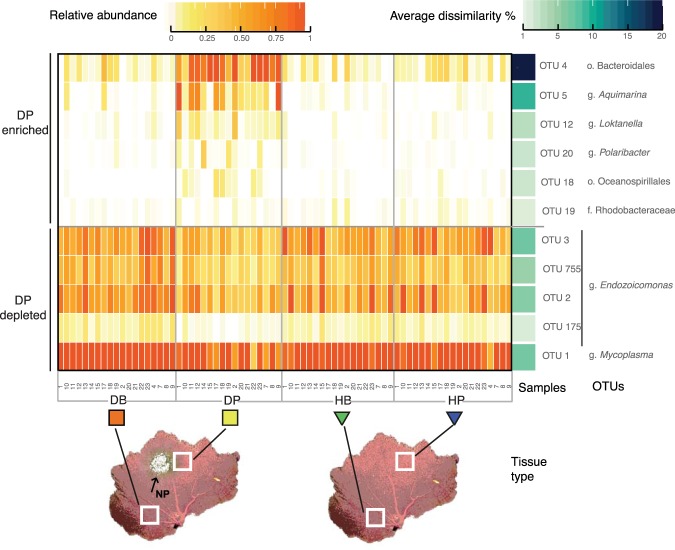
Heatmap of the relative abundances of OTUs in tissue samples from healthy and diseased colonies. According to the SIMPER analyses, the eleven OTUs depicted mainly contributed to the differentiation between symptomatic (DP) and asymptomatic samples (HB, HP, DB), (g: genus, f: family, c: class, o: order). Average dissimilarity (%) corresponds to OTU’s contribution to the dissimilarity between DP and the rest of samples (DB, HP and HB).

Considering all samples (n = 73), twenty-nine OTUs accounted for the cumulative 95% of total abundance. Eleven from these twenty-nine OTUs contributed to a greater extent to the differentiation between symptomatic (DP) and asymptomatic samples (DB, HP and HB), exhibiting two opposite trends: enrichment and depletion (Fig. 4). In terms of enrichment, a member from the order Bacteroidales (OTU4) was more abundant in DP samples than in the rest of asymptomatic samples and contributed the highest proportion to this differentiation (about 20%) (Fig. 4, Supplementary Table S3 and S4). In addition, OTUs from the genus *Aquimarina* (Flavobacteriaceae, OTU5) and the genus *Loktanella* (Rhodobacteraceae, OTU12) also displayed higher abundances in DP samples, contributing over 10% and about 4% to the differentiation, respectively. Relative abundances of representatives from the family Flavobacteriaceae (OTU19), the genus *Polaribacter* (Flavobacteriaceae, OTU20) and the order Oceanospirillales (OTU18) increased in DP samples, but contributed to a lesser extent to the dissimilarity (<3%). In contrast, the relative abundances of the *Endozoicomonas* and *Mycoplasma* OTUs, previously described as part of the core microbiome of the healthy colonies, decreased in DP samples and contributed between 1.70% and 8.80% to the dissimilarity with the rest of asymptomatic samples (Fig. 4, Supplementary Tables S3 and S4).

**Figure 5:**
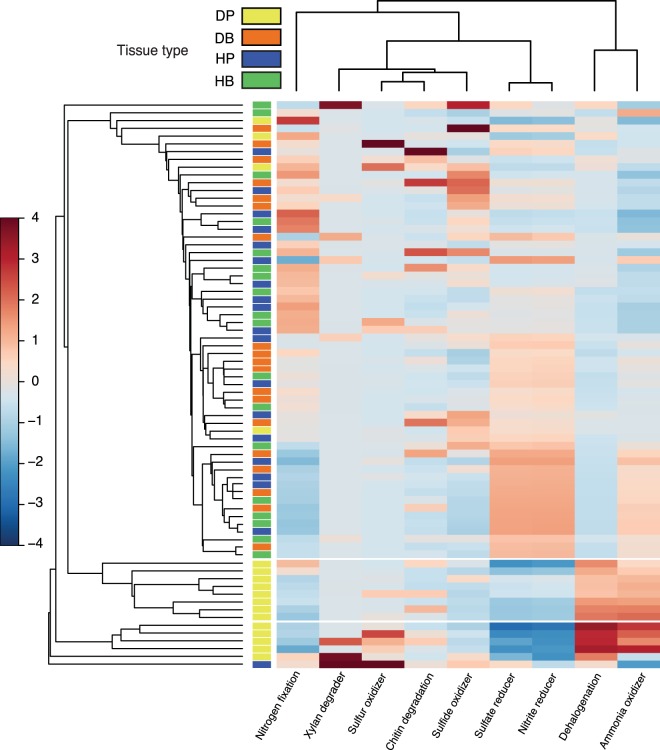
Taxonomy-based functional profiling of bacterial communities in samples from healthy and diseased colonies. Shifts in potential functional differences are represented by a relative abundance scale showing the enrichment (red colour) and depletion (blue colour) in different metabolic profiles mapped to the corresponding taxonomic information by METAGENassist. Hierarchical clustering of samples and functions was performed by a single linkage algorithm using Euclidean distance measurements.

In addition to shifts in bacterial community compositions, we also observed a different taxonomy-based functional profile in DP samples in comparison to the asymptomatic samples (HB, HP and DB). DP samples clustered in two groups enriched in bacterial taxa related to the physiological categories ‘anaerobic’, ‘heterotroph’, and the metabolic categories ‘dehalogenation’ and ‘ammonia oxidizer’. In contrast, these symptomatic samples displayed depletion in bacterial taxa linked to ‘nitrite reducer’ and ‘sulphate reducer’ metabolisms (Fig. 5, Supplementary Fig. S2). Interestingly, we observed functional diversity within healthy samples, although the bacterial community compositions did not differ significantly. Contrasting patterns regarding nitrogen fixers and sulphate and nitrite reducers were observed between clusters of mainly asymptomatic samples (Fig. 5).

### Characterisation of *Pacifigorgia cairnsi* disease lesions

Based on field observations, diseased colonies are characterized by necrotic patches (visible gorgonian axis without coenenchyme). Lesions are commonly located at the periphery of the colony and multifocally distributed. The shape of lesions is circular to irregular with undulating margins (*sensu*^25^). Moreover, lesions are typically surrounded by tissue with retracted polyps (Fig. 1). Based on these observations, we here refer to this newly reported disease as ‘necrotic patch disease’ (NPD). According to the microscopic pathology, no differences were observed among the four types of tissue. The coenenchyme from the surface and from longitudinal and transversal sections revealed the same microscopic irregular morphology in HB, HP, DP and DB. Additionally, no signs of fungal presence (i.e. hyphae and conidiophores) were observed in healthy or diseased tissues (Supplementary Fig. S3).

## Discussion

In this study, we tested NPD-related changes in the bacterial microbiome of *P*. *cairnsi* sea fans. Our major findings are (i) the core microbiome composition associated with healthy colonies is similar in basal and peripheral tissues, (ii) bacterial microbiome shifts in diseased tissues are driven by the decrease of potential endosymbionts composing the healthy core microbiome and the appearance of an opportunistic consortium, and (iii) the NPD-related consortium is confined to the symptomatic tissues of the affected colonies.

Bacterial communities constituting the core microbiome associated with healthy colonies did not differ significantly between basal and peripheral tissues. In terms of photosynthetic endosymbionts, different spatial arrangements have been observed within branching scleractinian corals as a consequence of small-scale environmental variability (e.g. different light intensity and water flow)^26^. Correlation between bacterial communities and microhabitats within the colony is not well known yet. We hypothesize that the flexible fan-shape, which allows uniform water flow through the mesh in order to maximize the filter-feeding efficiency^27^, may lead to the similarity of bacterial communities observed between peripheral and basal parts.

The bacterial core microbiome of the sea fans was dominated by members assigned to the order Oceanospirillales and the genus *Mycoplasma*. Four OTUs from Oceanospirillales belonged to the genus *Endozoicomonas*, being the most diverse taxonomic group of the core. *Endozoicomonas* are commonly found as dominant endosymbionts in scleractinian and gorgonian core microbiomes^28–31^. Multiple metabolic functions are attributed to this taxon within healthy coral holobionts such as gluconeogenesis, transport of molecules and synthesis of amino acids^32,33^. Additionally, *Endozoicomonas* spp. may play an important role in coral health by providing antimicrobial activity^34^. Particularly, *Endozoicomonas* and *Pseudovibrio* isolates from coral and sponges showed antagonistic effects against different bacterial groups including known coral pathogens as *Vibrio coralliilyticus*^10,34,35^.

The most abundant OTU in all samples was assigned to the genus *Mycoplasma*. This taxon is an common microbiome member in gorgonians^36,37^ and cold-water scleractinians^38,39^. Even though *Mycoplasma* spp. have been suggested to be harmless commensals or endosymbionts in corals and in some cnidarians^38–40^, their specific role within the coral holobiont remains unclear.

Overall, our data revealed that the taxa constituting the sea fan bacterial core microbiome are also abundant in other healthy gorgonians, such as the Mediterranean *Leptogorgia sarmentosa* and *Eunicella* spp.^14^, the Caribbean *Antillogorgia elisabethae*^41^ and the eastern Pacific *Muricea* spp.^37^. This indicates that these bacterial groups play similar roles in maintaining the holobiont’s health status. However, specific strains of *Mycoplasma* and *Endozoicomonas* have been associated with particular gorgonian species, potentially suggesting coevolutionary bacteria-host associations^28,37,42,43^. Hence, identifying the specific role that each of these potential endosymbionts play within their hosts might provide valuable insights to understand the relationships between gorgonians and their microbiomes.

Significant shifts in bacterial community compositions and functional profiles were observed between healthy-colony and NPD-affected tissues. The relative abundances of the main core members (i.e. *Endozoicomonas* and *Mycoplasma*) decreased in diseased tissues, while a different array of more diverse bacteria arose. The depletion of healthy-state associated taxa in conjunction with the increase of bacterial diversity, is not only common in corals affected by Yellow Band Disease (YBD)^20^, White Plague Disease (WPD)^44,45^ and Black Band Disease (BBD)^18^, but is also common in necrotic and unusual coral lesions^46,47^.

An OTU assigned to the order Bacteroidales contributed the most to the differentiation between healthy-colony and symptomatic tissues. Interestingly, this OTU corresponded to a low-abundant member of the *P*. *cairnsi* bacterial microbiome in the healthy tissues (Figs 3 and 4). Physiological or competitive constraints acting in healthy tissues might be eliminated during the disease, allowing native bacteria to increase opportunistically^46^. Additionally, the diseased tissues exhibited relatively high abundances of members of the families Flavobacteriaceae and Rhodobacteraceae, which have been associated with polymicrobial consortiums in BBD and WPD^18,45^. These taxa are considered opportunistic commensals capable of degrading the coral host tissue^18,44^. Specifically, *Aquimarina* spp. and *Polaribacter* spp. have been found in bleached marine macroalgae^48^ and degrading polymers in lobsters’ lesions^49^. Overall, the NPD-related consortium was comprised of opportunistic bacteria represented by i) native microbiome members at increased abundances and by ii) a group of bacteria with the potential ability for degrading the host tissue, persisting in a broad host-range.

Shifts in the bacterial community compositions were consistent with changes in the functional profiles between tissues from healthy colonies and symptomatic tissues. The most noteworthy switch was the decrease of sulphate and nitrite reducing taxa in diseased tissues. This shift may be related to the reduction of potential endosymbionts in symptomatic tissues since these metabolic functions have previously been identified in bacterial symbionts associated with healthy corals^50–52^. Although increased abundances of ammonia oxidizing bacteria were observed in some healthy samples, this metabolic function was remarkably increased in diseased tissues. The latter may respond to the higher availability of nitrogen compounds (particularly NH_3_) in symptomatic tissues, derived from organic matter decomposition^53^, which in turn may be related to the increase of anaerobic, heterotrophic, and dehalogenating taxa^54,55^. These findings, suggest that the NPD-related consortium likely uses diverse sources for energy, carbon and nutrient acquisition, suggesting that it opportunistically exploits available niches that may emerge as a consequence of the decrease of resident bacteria and the decay of coral tissue.

Additionally, we did not observe any evidence of fungal presence in HP, HB, DP and DB in our morphological assessment of tissues. Fungal species, such as *Aspergillus sydowii*, have previously been reported as causative agent of aspergillosis, a disease causing mass mortalities of *Gorgonia ventalina* sea fans in the Caribbean^56^. However, the role of *A*. *sydowii* as pathogen has been questioned^57,58^, as it has been found in healthy and diseased gorgonian octocorals in TEP (Tropical Eastern Pacific)^59,60^. Whether NPD affecting *P*. *cairnsi* is the same disease observed in other *Pacifigorgia* species in the TEP or whether it is aspergillosis, should be further addressed. However, we did not observe tissue purpling (i.e. host-produced melanin) as a response against fungal presence in any *P*. *cairnsi* sea fan, which has been considered to be a characteristic symptom of aspergillosis^61,62^. Therefore, the diagnoses of the disease here described (i.e. necrotic patches surrounded by tissue with retracted polyps and no microscopic evidence of fungal presence) lead us to consider NPD as a novel disease affecting gorgonian sea fans in the TEP.

Overall, our results suggest that disruptions in the natural bacterial community, depicted as a decrease of potential endosymbionts (e.g. *Endozoicomonas*), may favour the appearance of opportunistic taxa in diseased gorgonians. Correlation exists between coral health and the presence of endosymbionts, such as *Endozoicomonas* that are involved in nutrient acquisition and production of antimicrobial compounds^34,41,63^. Decreasing abundances of these endosymbionts are characteristic of diseased and anthropogenically impacted corals^64,65^, suggesting that imbalance in resident coral microbiota may have dramatic effects on coral health. The NPD-related consortium is likely in a state of microbial imbalance (i.e. dysbiosis)^66^, potentially driven by environmental pressures such as anomalous sea water temperatures^22^. In fact, it has been argued that many marine diseases may be the consequence of microbial dysbiosis and the rise of opportunistic or polymicrobial infections^18,46,67^ rather than being caused by single pathogens^67–69^.

The lack of differences in bacterial community compositions and functional profiles between tissues from healthy colonies and the asymptomatic tissues from diseased colonies suggests that the latter may be healthy. Hence, two health states could be identified within diseased colonies: (i) the diseased state of tissues affected by the NPD-related consortium and (ii) the healthy state of asymptomatic tissues colonized by a natural microbiome community. Our data thus suggest that the disease-related consortium is locally confined in the symptomatic tissues of *P*. *cairnsi*. This contrasts with findings in *Orbicella faveolata* affected by YBD, where asymptomatic tissues hosted microbial communities that differed both from symptomatic and healthy tissues, thus suggesting intermediate health states^20^.

The confinement of the disease-related consortium in *P*. *cairnsi* may also facilitate colony recovery. Corals showing localized BBD showed reduction of mortality rates as well as the halt of disease progression after disease treatment (i.e. removal of the affected area and sealing it with marine epoxy)^70^. However, diseased *P*. *cairnsi* sea fans have been seen recovering naturally after NPD incidences^21,22^. Breakages of both fragile NPD-affected areas and healthy tissue were observed in *P*. *cairnsi* due to the effect of strong currents (EQ pers. obs.). Thus, natural breaking offs of affected tissues may contribute to convalescence and ultimately to the resilience of these gorgonian populations.

Our study reveals shifts in the bacterial microbiome associated with a newly reported disease affecting tropical gorgonian corals. It recognizes the strong relationship between coral disease and shifts in the microbiome, and reveals the potential link between the spatial effects of the disease-related consortium at intra-colony level and disease recovery. Given the pivotal role that endosymbionts play in coral health status, future studies should focus on elucidating their specific functions within the holobiont, in order to better understand host-microbiome associations. Additionally, we encourage exploring the effect of environmental disturbances in microbiome disruptions triggering disease outbreaks, by implementing long-term studies and examining transcriptomic host profiles.

## Methods

### Sample collection

A total of 40 colonies (20 healthy and 20 diseased) of *Pacifigorgia cairnsi* sea fans were sampled around Malpelo, an oceanic remote island about 500 km off the Colombian coast in the Tropical Eastern Pacific (3°58′30″N, 81°34′48″W). All samples were collected on the same day between 10 and 15 m depth by Scuba diving at the ‘El Arrecife’ site. Sampled sea fans were adult colonies of approximately the same size in order to avoid age-related variations in microbial communities^71^. Healthy and diseased colonies were chosen based on the absence or presence of damaged tissues, respectively. From each colony two samples (5 × 5 cm) were taken, one from the periphery and one from the base, separated by at least 20 cm. Accordingly, 20 samples were obtained from each of the four types of tissues: peripheral tissue from healthy colonies (HP), basal tissue from healthy colonies (HB), peripheral (symptomatic) tissue from diseased colonies (DP) and basal (but asymptomatic) tissue from diseased colonies (DB) (Fig. 1). The actual necrotic parts consisting of dead gorgonian axis (without coenenchyme) were not included in the symptomatic tissue samples but only the surrounding area of the wounds (Fig. 1). In order to prevent sample contamination, all wearing gloves and tools used to collect or manipulate samples were either disposed or sterilized after single use. Furthermore, each sample was gently rinsed with 100 ml filtered fresh water in order to remove exogenous or transient microorganisms loosely associated with the coral tissue. Samples were stored in RNAlater (Thermo Fisher Scientific, Waltham, USA) and preserved at −80 °C until subsequent DNA extraction.

Collections were made possible with research permit No.105 (2013), issued by the Autoridad Nacional de Licencias Ambientales-ANLA, Ministerio de Ambiente y Desarrollo Sostenible, Colombia and Contrato de Acceso a Recursos Genéticos para Investigación Científica Sin Interés Comercial No. 106, 20 (2014) RGE0114.

### DNA extraction and 16S rRNA gene sequencing

DNA was extracted from approximately 100 mg of sea fan tissue using the PowerSoil DNA Isolation Kit (Mo Bio Laboratories, Carlsbad, USA) after macerating the sample in liquid nitrogen. DNA was initially quantified using a Nanodrop 2000 UV-Bis Spectrophotometer (Thermo Fisher Scientific) and corroborated prior to sequencing with a Qubit fluorometer HS assay kit (Life Technologies, Carlsbad, USA).

The variable V4 region of the 16S rRNA gene was sequenced using the 515 F/806 R PCR primers and Illumina flowcell adapter sequences according to the earth microbiome protocol^72^ (http://www.earthmicrobiome.org/emp-standard-protocols/16s/). The Takara Taq DNA polymerase premix was used for PCR amplifications as described by Pehrsson *et al*.^73^. Barcoded amplicons were pooled and sequenced on the Illumina MiSeq platform (Illumina, San Diego, USA), implementing 2 × 250 bp paired-end read libraries.

### Data pre-processing and OTU picking

Sequenced reads were pre-processed and Operational Taxonomic Units (OTUs) were generated following the UPARSE pipeline^74^ with the modifications suggested by Gibson *et al*.^75^ and Pehrsson *et al*.^73^. In brief, reads were de-multiplexed using *split_libraries_fastq*.*py* script included in QIIME v1.9.1^76^. Then, paired-end reads were processed by USEARCH v8.1.1861^77^ as follows: merged (*usearch–fastq_mergepairs*) requiring a final length of 253 bp ± 5 bp, quality filtered (*usearch–fastq_filter*) allowing a maximum expected error of 0.5, dereplicated (*usearch–derep*), sorted excluding singletons (*usearch–sortbysize*), clustered in OTUs (*usearch–cluster_otus*) and checked for chimeras (*usearch–uchime_ref)* using the ChimeraSlayer gold database (v. microbiomeutil-r20110519, downloaded in May 2016). Reads were later mapped to OTUs and their assignment was performed at 97% identity threshold (*usearch–usearch_global*). OTUs were aligned using PyNAST^78^ and taxonomy was assigned by RDP classifier^79^ against the GreenGenes database^80^ as implemented by QIIME scripts (*align_seqs*.*py*, *assign_taxonomy*, *filter_alignment*.*py*). Assigned taxonomy was synchronized with OTUs in Biom format tables^81^. The complete dataset has been deposited in the NCBI Sequence Read Archive (SRA) under BioProject number PRJNA403829.

In order to obtain a comprehensive description of the within-sample bacterial community, alpha diversity metrics (total number of observed OTUs, species richness Chao1 and Shannon diversity) and rarefaction plots with 29,000 sequences per sample were generated through QIIME (*alpha_rarefaction*.*py*, *single_rarefaction*.*py*). Differences in alpha diversity metrics were tested between all pairs of tissue types with Kruskal-Wallis tests as implemented in the software PAST v3.12^82^. The significance level was adjusted for the number of comparisons tested by Bonferroni correction.

### Bacterial community analyses

Multivariate analyses were conducted to assess differences in bacterial community compositions between samples (beta diversity): (i) from healthy colonies (HD and HP) and ii) from healthy and diseased colonies (HP, HD, DP and DB). In both cases we considered taxa accounting for the cumulative 95% of total abundance. In order to visualize differences in bacterial community compositions between samples from tissue types we performed principal coordinate analyses (PCoA), applying a square-root transformation to relative abundances and calculating Bray-Curtis dissimilarity matrices. Permutational Analyses of Variance (PERMANOVA^83^) were conducted to test differences in bacterial community compositions between tissue types (9999 permutations). Additionally, Permutational Analyses of Multivariate Dispersions (PERMDISP^24^) were used to test for homogeneity of multivariate dispersions (9999 permutations) between sampling groups. The significance level was adjusted for the number of comparisons tested by Bonferroni correction. Similarity Percentage (SIMPER) analyses were used to identify the taxa contributing to the greatest extent to the observed patterns. All multivariate analyses were performed at family and OTU level using PRIMER 6 & PERMANOVA+ software^84^. Additionally, heatmaps were generated with the R package ggplot2^85,86^ through the RStudio suite^87^ to visualize patterns of similarity in OTUs’ abundances between types of tissues. As all samples were taken from the same reef and at the same day, we defined the core bacterial microbiome of *P*. *cairnsi* as those OTUs present in 100% of HB and HP sample tissues^14^.

Putative functional differences associated with differences in bacterial community compositions among tissue types were assessed by using METAGENassist^88^. OTUs filtering and normalization parameters were used as described by Hadaidi *et al*.^89^. Euclidean distance measure (single linkage algorithm) was used to visualize functional profiles (i.e. metabolism, oxygen requirements, carbon and energy source) in heatmaps mapped to the microbial communities.

### Characterisation of *Pacifigorgia cairnsi* disease lesions

In order to provide a detailed description of disease lesions, we conducted field investigations based on *in situ* underwater photographs from healthy and affected colonies.

This information was used subsequently to characterize and name the *P*. *cairnsi* disease as ‘necrotic patch disease’ according to a framework systematically describing gross lesions in corals^25^.

Moreover, the microscopic pathology of lesions was addressed using scanning electron microscopy (SEM, JSM 6490-LV)^90^. Accordingly, HP, HB, DP and DB tissues from five healthy and five diseased colonies were fixed in 2% glutaraldehyde, washed in distilled sterile water, dehydrated in a series of ethanol solutions (30, 50, 70, 80, 90, 95 and 100%) and finally carbon coated. SEM images were obtained from the surface and from longitudinal and transversal sections of all types of tissues at 45, 250 and 1000 x magnifications. Finally, detailed observations of polyps, coenenchymes and gorgonian axes were done using SEM at up to 2000x magnification.

## Electronic supplementary material

Supplementary Information
