# Supplementary material for: Local confinement of disease-related microbiome facilitates recovery of gorgonian sea fans from necrotic-patch disease
Source: Sci Rep. 2018 Oct 2;8:14636. doi: 10.1038/s41598-018-33007-8 (PMC6168572; doi:10.1038/s41598-018-33007-8)
Supplement: Supplementary file 1 — Supplementary Information [file 41598_2018_33007_MOESM1_ESM.docx]

*Supplementary Figures & Tables*

*for*

**Local confinement of disease-related microbiome facilitates recovery of gorgonian sea fans from necrotic-patch disease**

Elena Quintanilla, Catalina Ramírez-Portilla, Boahemaa Adu-Oppong, Gretchen Walljasper, Stefanie P Glaeser, Thomas Wilke, Alejandro Reyes Muñoz, Juan A Sánchez

**Supplementary Figure S1** Rarefaction curves of (a) Chao 1 index, (c) number of observed OTUs and (e) Shannon diversity Average and standard deviation from 10 random subsamplings are shown. Kruskal-Wallis tests of (b) Chao 1 index, (d) number of observed OTUs and (f) Shannon diversity between samples from tissue types at a subsampling of 29,000 reads per sample. **P*<0.0083, corrected Bonferroni. DP: diseased peripheral (symptomatic), DB: diseased basal (asymptomatic), HP: healthy peripheral and HB: healthy basal. The number of samples from each type of tissue is indicated within parenthesis.

**Supplementary Figure S2** Taxonomy-based functional profile of bacterial communities in samples from healthy and diseased colonies. Shifts in potential functional differences according to (a) oxygen requirement and (b) carbon source, are represented in a relative scale showing the enrichment (red colour) and depletion (blue colour). Clustering of samples and functions was performed by Euclidean distance measure (single linkage algorithm). DP: diseased peripheral, DB: diseased basal, HP: healthy peripheral and HB: healthy basal.

**Supplementary Figure S3** Scanning electron microscopy images showing healthy and diseased tissues of *Pacifigorgia cairnsi*. (a) Details of polyps and coenenchyme of HP tissue surface. (b) Longitudinal section of HB tissue showing the axis and coenenchyme. Details of coenenchyme from longitudinal section of (c) HP, (d) DP, (e) HB and (f) DB tissues. Polyps (1), coenenchyme (2), and axis (3). The circle shows the area from which images (c), (d), (e) and (f) were taken.

| Tissue type | n | Alpha diversity metrics (±SD) | | |
| --- | --- | --- | --- | --- |
|  |  | Chao1 | Shannon | Observed OTUs |
| DP | 17 | 248.18 ± 93 | 3.12 ± 0.72 | 212.53 ± 83 |
| DB | 19 | 137.60 ± 68 | 2.56 ± 0.29 | 115.26 ± 55 |
| HP | 19 | 150.73 ± 49 | 2.59 ± 0.55 | 133.05 ± 44 |
| HB | 18 | 110.61 ± 58 | 2.47 ± 0.51 | 97.27 ± 52 |

**Supplementary Table S1** Overview of alpha diversity metrics (average ± SD) of samples from each type of tissue. Metrics were calculated at an even subsampling of 29,000 reads per sample. DP: diseased peripheral, DB: diseased basal, HP: healthy peripheral, HB: healthy basal and n: number of samples.

| OTUs | Average Abundance | Average Similarity | Standard deviation | Contribution (%) | Cumulative (%) |
| --- | --- | --- | --- | --- | --- |
| Group HB |  |  |  |  |  |
| (Average similarity 79.12) |  |  |  |  |  |
| OTU1_*Mycoplasma* | 6.67 | 24.24 | 6.67 | 30.64 | 30.64 |
| OTU3_*Endozoicomonas* | 4.2 | 14.7 | 5.6 | 18.58 | 49.22 |
| OTU2_*Endozoicomonas* | 4.07 | 13.67 | 4.66 | 17.28 | 66.5 |
| OTU755_*Endozoicomonas* | 2.92 | 10.14 | 4.14 | 12.82 | 79.32 |
| OTU6_Oceanospirillaceae | 1.41 | 4.19 | 2.94 | 5.29 | 84.61 |
| OTU8_Bacteria | 1.07 | 3.46 | 3.48 | 4.38 | 88.99 |
| OTU175_*Endozoicomonas* | 0.68 | 2.33 | 3.84 | 2.95 | 91.94 |
| Group HP |  |  |  |  |  |
| (Average similarity: 77.79) |  |  |  |  |  |
| OTU1_*Mycoplasma* | 6.55 | 23.21 | 4.88 | 29.84 | 29.84 |
| OTU3_*Endozoicomonas* | 4.43 | 14.89 | 5.06 | 19.14 | 48.97 |
| OTU2_*Endozoicomonas* | 4.11 | 13.64 | 3.98 | 17.53 | 66.5 |
| OTU755_*Endozoicomonas* | 2.56 | 8.4 | 3.25 | 10.8 | 77.3 |
| OTU6_Oceanospirillaceae | 1.3 | 3.99 | 3.32 | 5.12 | 82.42 |
| OTU8_Bacteria | 0.9 | 2.81 | 2.93 | 3.62 | 86.04 |
| OTU4_Bacteroidales | 0.96 | 2.1 | 1.25 | 2.69 | 88.73 |
| OTU175_*Endozoicomonas* | 0.61 | 2.06 | 3.75 | 2.64 | 91.38 |

**Supplementary Table S2** Similarity Percentage analysis (SIMPER). Main OTUs driving similarities in bacterial community compositions within tissue from healthy colonies (HP and HB). HP: healthy peripheral, HB: healthy basal. Results explained >90% of the similarity observed.

| OTUs | DP | DB | HB | HP |
| --- | --- | --- | --- | --- |
| OTU4_Bacteroidales | 25 (± 15.00) | 1.19 (± 2.53) | 0.99 (± 1.52) | 1.39 (± 1.75) |
| OTU5_*Aquimarina* | 8.75 (± 11.11) | 0.20 (± 0.60) | 0.040 (± 0.06) | 0.03 (± 0.06) |
| OTU12_*Loktanella* | 1.29 (± 1.80) | 0.05 (± 0.08) | 0.04 (± 0.09) | 0.043 (± 0.09) |
| OTU20_*Polaribacter* | 0.82 (± 1.65) | 0.02 (± 0.50) | 0.013 (± 0.02) | 0.01 (± 0.02) |
| OTU18_Oceanospirillales | 0.51 (± 0.76) | 0.01 (± 0.02) | 0.01 (± 0.03) | 0.05 (± 0.13) |
| OTU19_Rhodobacteraceae | 0.20 (± 0.18) | 0.03 (± 0.04) | 0.09 (± 0.19) | 0.10 (± 1.50) |
| OTU3_*Endozoicomonas* | 7.63 (± 4.20) | 17.36 (± 5.65) | 18.22 (± 8.07) | 20.71 (± 11.83) |
| OTU755_*Endozoicomonas* | 4.51(± 2.99) | 11.95 (± 5.64) | 8.92 (± 3.96) | 7.00 (± 3.60) |
| OTU2_*Endozoicomonas* | 10.056 (± 5.77) | 19.74 (± 5.36) | 17.57 (± 9.77) | 18.00 (± 10.14) |
| OTU175_*Endozoicomonas* | 0.27 (± 0.20) | 0.69 (± 0.30) | 0.49 (± 0.23) | 0.40 (± 0.19) |
| OTU1_*Mycoplasma* | 33.61 (± 16.12) | 39.81 (± 9.24) | 45.08 (± 13.04) | 43.82 (± 14.34) |

**Supplementary Table S3** Relative abundances (average ± SD) of OTUs that contributed to a greater extent to the differentiation between symptomatic (DP) and asymptomatic samples (HB, HP, DB). HP: healthy peripheral, HB: healthy basal, DP: diseased peripheral and DB: diseased basal.

| OTUs | Average Abundance | Average Abundance | Average Dissimilarity | Standard deviation | Contribution (%) | Cumulative (%) |
| --- | --- | --- | --- | --- | --- | --- |
| Groups DP & DB | Group DP | Group DB |  |  |  |  |
| (Average dissimilarity: 37.06) |  |  |  |  |  |  |
| OTU4_Bacteroidales | 4.96 | 0.77 | 7.51 | 2.18 | 20.25 | 20.25 |
| OTU5_*Aquimarina* | 2.35 | 0.21 | 3.79 | 1.31 | 10.22 | 30.48 |
| OTU3_*Endozoicomonas* | 2.64 | 4.11 | 2.69 | 1.58 | 7.27 | 37.75 |
| OTU755_*Endozoicomonas* | 1.95 | 3.37 | 2.64 | 1.54 | 7.12 | 44.87 |
| OTU2_*Endozoicomonas* | 3.02 | 4.4 | 2.6 | 1.53 | 7.01 | 51.88 |
| OTU1_*Mycoplasma* | 5.49 | 6.27 | 2.58 | 1.38 | 6.95 | 58.83 |
| OTU12_*Loktanella* | 0.95 | 0.14 | 1.42 | 1.41 | 3.83 | 62.66 |
| OTU7_Alteromonadales | 0.51 | 0.97 | 1.22 | 0.85 | 3.3 | 65.96 |
| OTU6_Oceanospirillaceae | 1.05 | 1.51 | 1.18 | 1.23 | 3.19 | 69.15 |
| OTU18_Oceanospirillales | 0.59 | 0.04 | 0.98 | 1.22 | 2.66 | 71.81 |
| OTU20_*Polaribacter* | 0.63 | 0.09 | 0.98 | 0.9 | 2.65 | 74.46 |
| OTU8_Bacteria | 0.68 | 0.99 | 0.85 | 1.4 | 2.28 | 76.74 |
| OTU10_Alphaproteobacteria | 0.49 | 0.03 | 0.83 | 0.32 | 2.24 | 78.99 |
| OTU17_Kiloniellales | 0.51 | 0.27 | 0.8 | 0.91 | 2.15 | 81.14 |
| OTU9_Kiloniellales | 0.12 | 0.43 | 0.78 | 0.43 | 2.12 | 83.25 |
| OTU13_Bacteria | 0.44 | 0.43 | 0.75 | 1.26 | 2.03 | 85.29 |
| OTU15_Alphaproteobacteria | 0.22 | 0.43 | 0.71 | 1.06 | 1.93 | 87.21 |
| OTU175_*Endozoicomonas* | 0.48 | 0.81 | 0.63 | 1.68 | 1.7 | 88.92 |
| OTU14_*Pirellulaceae* | 0.2 | 0.17 | 0.54 | 0.59 | 1.47 | 90.39 |
| Groups DP & HP | Group DP | Group HP |  |  |  |  |
| (Average dissimilarity: 36.79) |  |  |  |  |  |  |
| OTU4_Bacteroidales | 4.96 | 0.95 | 7.19 | 2.07 | 19.54 | 19.54 |
| OTU5_*Aquimarina* | 2.35 | 0.11 | 3.89 | 1.34 | 10.58 | 30.12 |
| OTU3_*Endozoicomonas* | 2.64 | 4.4 | 3.23 | 1.41 | 8.79 | 38.92 |
| OTU1_*Mycoplasma* | 5.49 | 6.52 | 3.06 | 1.41 | 8.32 | 47.23 |
| OTU2_*Endozoicomonas* | 3.02 | 4.09 | 2.54 | 1.31 | 6.91 | 54.14 |
| OTU755_*Endozoicomonas* | 1.95 | 2.55 | 1.7 | 1.46 | 4.63 | 58.77 |
| OTU12_*Loktanella* | 0.95 | 0.12 | 1.46 | 1.46 | 3.97 | 62.74 |
| OTU20_*Polaribacter* | 0.63 | 0.08 | 0.98 | 0.9 | 2.68 | 65.41 |
| OTU11_Alphaproteobacteria | 0.05 | 0.52 | 0.94 | 0.53 | 2.56 | 67.97 |
| OTU10_Alphaproteobacteria | 0.49 | 0.12 | 0.93 | 0.37 | 2.52 | 70.49 |
| OTU18_Oceanospirillales | 0.59 | 0.13 | 0.91 | 1.18 | 2.46 | 72.95 |
| OTU6_Oceanospirillaceae | 1.05 | 1.29 | 0.9 | 1.35 | 2.44 | 75.39 |
| OTU17_Kiloniellales | 0.51 | 0.48 | 0.86 | 1.09 | 2.35 | 77.73 |
| OTU13_Bacteria | 0.44 | 0.61 | 0.84 | 1.28 | 2.28 | 80.01 |
| OTU7_Alteromonadales | 0.51 | 0.66 | 0.8 | 1.01 | 2.17 | 82.18 |
| OTU14_Pirellulaceae | 0.2 | 0.26 | 0.68 | 0.57 | 1.85 | 84.03 |
| OTU8_Bacteria | 0.68 | 0.9 | 0.67 | 1.29 | 1.83 | 85.86 |
| OTU9_Kiloniellales | 0.12 | 0.38 | 0.67 | 0.66 | 1.81 | 87.67 |
| OTU15_Alphaproteobacteria | 0.22 | 0.3 | 0.59 | 1.08 | 1.61 | 89.28 |
| OTU22_*Synechococcus* | 0.24 | 0.38 | 0.55 | 0.7 | 1.51 | 90.79 |
| Groups DP & HB | Group DP | Group HB |  |  |  |  |
| (Average dissimilarity: 37.59) |  |  |  |  |  |  |
| OTU4_Bacteroidales | 4.96 | 0.71 | 7.68 | 2.17 | 20.44 | 20.44 |
| OTU5_*Aquimarina* | 2.35 | 0.14 | 3.88 | 1.33 | 10.34 | 30.78 |
| OTU1_*Mycoplasma* | 5.49 | 6.64 | 3.04 | 1.4 | 8.09 | 38.86 |
| OTU3_*Endozoicomonas* | 2.64 | 4.18 | 2.89 | 1.5 | 7.68 | 46.55 |
| OTU2_*Endozoicomonas* | 3.02 | 4.05 | 2.48 | 1.28 | 6.59 | 53.13 |
| OTU755_*Endozoicomonas* | 1.95 | 2.91 | 2.05 | 1.47 | 5.45 | 58.59 |
| OTU12_*Loktanella* | 0.95 | 0.1 | 1.52 | 1.49 | 4.04 | 62.62 |
| OTU6_Oceanospirillaceae | 1.05 | 1.4 | 1.07 | 1.25 | 2.84 | 65.46 |
| OTU20_*Polaribacter* | 0.63 | 0.06 | 1.02 | 0.92 | 2.72 | 68.18 |
| OTU18_Oceanospirillales | 0.59 | 0.05 | 0.98 | 1.21 | 2.62 | 70.8 |
| OTU13_Bacteria | 0.44 | 0.61 | 0.98 | 1.03 | 2.61 | 73.41 |
| OTU10_Alphaproteobacteria | 0.49 | 0.06 | 0.87 | 0.33 | 2.32 | 75.73 |
| OTU8_Bacteria | 0.68 | 1.06 | 0.84 | 1.38 | 2.24 | 77.97 |
| OTU17_Kiloniellales | 0.51 | 0.18 | 0.84 | 0.87 | 2.24 | 80.21 |
| OTU7_Alteromonadales | 0.51 | 0.6 | 0.74 | 1.06 | 1.96 | 82.17 |
| OTU9_Kiloniellales | 0.12 | 0.36 | 0.66 | 0.69 | 1.75 | 83.93 |
| OTU15_Alphaproteobacteria | 0.22 | 0.36 | 0.64 | 1.25 | 1.7 | 85.63 |
| OTU16_34P16 | 0.05 | 0.32 | 0.64 | 0.35 | 1.69 | 87.32 |
| OTU14_Pirellulaceae | 0.2 | 0.22 | 0.6 | 0.56 | 1.61 | 88.93 |
| OTU19_Rhodobacteraceae | 0.41 | 0.19 | 0.57 | 1.66 | 1.51 | 90.44 |

**Supplementary Table S4** Similarity Percentage analysis (SIMPER). Main OTUs driving differences in bacterial community compositions between DP samples and the rest of tissue samples (DB, HB and HP). HP: healthy peripheral, HB: healthy basal, DP: diseased peripheral and DB: diseased basal. Results explained >90% of the dissimilarity observed.
